# Recommendations for terminology and the identification of neuropathic pain in people with spine-related leg pain. Outcomes from the NeuPSIG working group

**DOI:** 10.1097/j.pain.0000000000002919

**Published:** 2023-05-25

**Authors:** Annina B. Schmid, Brigitte Tampin, Ralf Baron, Nanna B. Finnerup, Per Hansson, Aki Hietaharju, Kika Konstantinou, Chung-Wei Christine Lin, John Markman, Christine Price, Blair H. Smith, Helen Slater

**Affiliations:** a Nuffield Department of Clinical Neurosciences, University of Oxford, Oxford, United Kingdom; b Department of Physiotherapy, Sir Charles Gairdner Hospital, Perth, Australia; c Curtin School of Allied Health, Faculty of Health Sciences, Curtin University, Perth, Australia; d Faculty of Business and Social Sciences, Hochschule Osnabrueck, University of Applied Sciences, Osnabrueck, Germany; e Division of Neurological Pain Research and Therapy, Department of Neurology, University Medical Center Schleswig-Holstein, Kiel, Germany; f Danish Pain Research Center, Department of Clinical Medicine, Aarhus University, Aarhus, Denmark; g Department of Neurology, Aarhus University Hospital, Aarhus, Denmark; h Department of Pain Management & Research, Norwegian National Advisory Unit on Neuropathic Pain, Division of Emergencies and Critical Care, Oslo University Hospital, Oslo, Norway; i Department of Molecular Medicine and Surgery, Karolinska Institutet, Stockholm, Sweden; j Department of Neurology, Tampere University Hospital, Tampere, Finland; k Faculty of Medicine and Health Technology, Tampere University, Tampere, Finland; l School of Medicine, Keele University, Keele, Staffordshire, United Kingdom; m Haywood Hospital, Midlands Partnership Foundation NHS Trust, Staffordshire, United Kingdom; n Institute for Musculoskeletal Health, The University of Sydney and Sydney Local Health District, Sydney, Australia; o Sydney Musculoskeletal Health, the University of Sydney, Sydney Australia,; p Translational Pain Research Program, Departments of Neurosurgery and Neurology, University of Rochester, Rochester, NY, United States; q Patient Advocate Nuffield Department of Clinical Neurosciences, University of Oxford, Oxford, United Kingdom,; r Division of Population Health and Genomics, University of Dundee, Dundee, Scotland; s enAble Institute, Faculty of Health Sciences, Curtin University, Perth, Australia

**Keywords:** sciatica, neuropathic pain, neuropathic pain grading scale, somatic referred pain, radiculopathy, radicular pain, spine-related leg pain

## Abstract

Pain radiating from the spine into the leg is commonly referred to as “sciatica,” “Sciatica” may include various conditions such as radicular pain or painful radiculopathy. It may be associated with significant consequences for the person living with the condition, imposing a reduced quality of life and substantial direct and indirect costs. The main challenges associated with a diagnosis of “sciatica” include those related to the inconsistent use of terminology for the diagnostic labels and the identification of neuropathic pain. These challenges hinder collective clinical and scientific understanding regarding these conditions. In this position paper, we describe the outcome of a working group commissioned by the Neuropathic Pain Special Interest Group (NeuPSIG) of the International Association for the Study of Pain (IASP) which was tasked with the following objectives: (1) to revise the use of terminology for classifying spine-related leg pain and (2) to propose a way forward on the identification of neuropathic pain in the context of spine-related leg pain. The panel recommended discouraging the term “sciatica” for use in clinical practice and research without further specification of what it entails. The term “spine-related leg pain” is proposed as an umbrella term to include the case definitions of somatic referred pain and radicular pain with and without radiculopathy. The panel proposed an adaptation of the neuropathic pain grading system in the context of spine-related leg pain to facilitate the identification of neuropathic pain and initiation of specific management in this patient population.

## Abstract 2

Supplemental Digital Content is Available in the Text.

## 1. Introduction

Pain radiating from the spine into the leg is commonly referred to as “sciatica.” “Sciatica” may be associated with significant consequences for the person living with the condition, imposing a reduced quality of life^35^ and substantial direct and indirect costs (eg, £1.2-10.6 billion annually in the United Kingdom alone, extrapolated from primary care data^34^).

Of note, there is no consensus on the definition and diagnostic criteria for “sciatica.” The term “sciatica” has been used to encompass a range of nerve-related conditions originating from the spine such as radicular pain or painful radiculopathy.^19,43^ Despite the linguistic allusion to neural involvement, “sciatica” has in some instances even been used to encompass somatic referred pain.^43^

Although these pain conditions may overlap, they are discrete entities with differing dominant pain characteristics (eg, nociceptive or neuropathic), which may require specific management approaches. Nevertheless, these terms are often used interchangeably, and these heterogeneous conditions are often amalgamated in clinical trials. Whether or not this amalgamation contributes to the modest treatment effects seen for “sciatica” is unclear.

Two of the main challenges associated with a diagnosis of “sciatica” relate to the inconsistent use of terminology for the diagnostic labels, “sciatica” or radicular pain or painful radiculopathy, and the identification of neuropathic pain. These challenges hinder collective clinical and scientific understanding and clarity regarding these conditions, impact effective clinical communication and care planning, prevent clear interpretation of the scientific literature related to the condition, and ultimately may contribute to the limited efficacy of care for people living with “sciatica.” Indeed, most trials on conservative management for people with “sciatica” either show no, or at best, modest effects.^11,25,31,32,42,44,46,47,58^

Here, we describe the outcome of a working group commissioned by the Neuropathic Pain Special Interest Group (NeuPSIG) of the International Association for the Study of Pain (IASP) and tasked with the following objectives:

(1) To revise the use of terminology for classifying spine-related leg pain (spine-related leg pain is an umbrella term that includes nerve-related [eg, radicular pain and painful radiculopathy] as well as somatic referred pain. As such, this term is broader than “sciatica,” which linguistically refers to neural involvement. The term “spine-related leg pain” is supported by the working group and was confirmed in the NeuPSIG membership consultation. For further details, see 3.3),
(2) To propose a way forward on the identification of neuropathic pain in the context of spine-related leg pain.

## 2. Methods

This working group was commissioned by NeuPSIG. The facilitators (A.B.S., B.T., and H.S.) were tasked with convening an international expert group with diverse expertise in the area of “sciatica,” neuropathic pain, and diagnostic grading.

We adopted a phased approach to address the objectives:

Phase 1a: discussion to reach consensus on proposed terminologies in the working group,
Phase 1b: consultation with the NeuPSIG membership to gauge members' preferences on the terminology proposition,
Phase 2: how to identify the presence of neuropathic pain in people presenting with spine-related leg pain (discussion to reach consensus in the working group).

### 2.1. Panel recruitment

Experts were identified by the facilitators in collaboration with the NeuPSIG management committee. Experts were required to have made substantial clinical, academic, or advocacy contributions to the field of “sciatica,” neuropathic pain, and diagnostic grading. Nine experts were invited by email, and all agreed to be a part of the working group. The 9 experts and 3 facilitators who formed the membership of the working group included 6 physicians, 5 physiotherapists, and 1 patient advocate (58% female). Specialities included musculoskeletal health (n = 5), neurology/pain specialist (n = 4), general practice (n = 1), neurosurgery (n = 1), and patient advocacy (n = 1). Most members (n = 9) had combined clinical and research roles with a median experience of 33.5 years (interquartile range 20.0) in clinical practice and 20 years (18.3) in research. The clinical settings of practising members were primary care (n = 2), secondary care (n = 1), and tertiary care (n = 5), with one expert working at the interface of primary and secondary care systems. Eight of the practicing experts were consulted with between 1 and 5 patients with spine-related leg pain per week, and one expert saw >10 per week. Our patient partner has lived experience of persistent “sciatica” with neuropathic leg pain and has extensive expertise with patient advocacy including communication with patients, educators, clinicians, academics, and professional associations.

The proposed recommendations on terminology in this paper were based on a thorough review of the literature as well as the results of an online survey designed to gather a basic understanding of experts' perspectives (see Appendix 1, available as supplemental digital content at http://links.lww.com/PAIN/B813). This was followed by 2 virtual meetings of 3 hours' duration, each conducted through Zoom. These replaced the planned face-to-face meeting during the IASP World Congress in Amsterdam 2021 which was cancelled due to COVID-19 restrictions. The working group members were briefed and updated about the progress before, between, and after the meetings with summary papers outlining the objectives, tasks, and outcomes of each workshop. Below we will elaborate on the challenges discussed and the resulting recommendations of the working group.

## 3. Phase 1: terminology

### 3.1. The conundrum of “sciatica”: setting the scene

**Figure 1.**
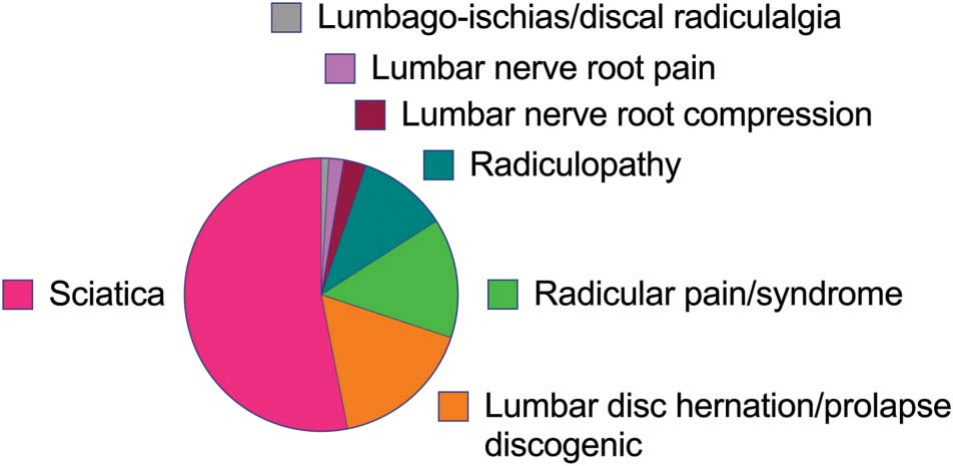
Terminology used in clinical trials to describe the study population with spine-related leg pain. Adapted from Lin et al.^43^

A recent systematic review demonstrated that multiple terms are used to describe spine-related leg pain. The most commonly used term is “sciatica” (Fig. 1).^43^ The definition of “sciatica” according to the Concise Oxford Medical Dictionary is “pain radiating from the buttock into the thigh, calf, and occasionally the foot.” This definition is immediately followed by the disclaimer “although it is in the distribution of the sciatic nerve, sciatica is rarely due to disease of this nerve.” This disclaimer highlights the controversy surrounding the term “sciatica.” It has been argued for many years that “sciatica” is an archaic term.^13,30^

Historically, the term “sciatica” was likely first introduced by Hippocrates and is derived from the Greek “ischios” for hip.^28^ Indeed, this term was originally used to describe pain in the pelvis and leg which was attributed to a diseased or subluxated hip. Since then, its underlying pathology has been assigned to a multitude of aetiologies, including abscesses, gout, and even turgidity of veins.^56^ In 1764, Domenico Cotugno of Naples finally distinguished “arthritic sciatica” from “nervous sciatica.”^56^ In the 19th century, “fractured” (herniated) discs were first described based on autopsies^59^ and mechanosensitivity of the nerve was mentioned in subsequent work by Lasegue and his student JJ Forst.^18,39^ In 1933, Mixter and Barr^50^ related “sciatica” to a herniated or prolapsed disc “encroaching” on the nerve root, which provided justification for surgical treatments.

Although some attempts have been made to better define spine-related leg pain,^10,23,67^ there is no consensus on terminology nor on the clinical criteria used to define “sciatica,” or different subgroups of spine-related leg pain in clinical trials.^43^ This conundrum has resulted in inconsistent criteria and their application to define spine-related leg pain in interventional studies.^22,43^

The absence of agreement on terminology and lack of consensus on case definition is likely a reason for the large range in prevalence data reported for “sciatica” (1.6%-43%).^36^ Importantly, the heterogeneity of patient populations resulting from the inconsistent use of terminology (including case definitions) and variable eligibility criteria may in part explain the conflicting evidence and influence the interpretation of efficacy of treatments (eg, physiotherapeutic interventions and pharmaceutical management) for people with “sciatica.”^11,12,32,43,47,58^

The IASP has, therefore, suggested that the term “sciatica” should not be used.^29^ Instead, they recommended that pain in the lower limb which originates from the lower back should be described with more precise case definitions such as referred pain, radicular pain, or (painful) radiculopathy.^30^ These definitions are in line with the terminology described by Bogduk et al.^6^ (Table 1).

**Table 1:** Definitions of specific types of spine-related leg pain and radiculopathy first described by Bogduk et al.^6^ and IASP.^30^

Terminology	Source	Characteristics	Distribution
Somatic-referred pain	Noxious stimulation of nerve endings in somatic structures (eg, discs, facet joints, muscles, **tendons, ligaments, fascia, and bones**)	Dull, aching, gnawing, and pressure	**Perceived at a location other than the site of the noxious stimulation**. Wide area, difficult to localise, and nondermatomal. Can be gluteal area, thigh, occasionally in lower leg, **rarely in the foot**. **Usually deep and rarely cutaneous**
Radicular pain (with or without radiculopathy, see definition below)	**Hyperexcitability and** ectopic discharges of dorsal roots or dorsal root ganglia (caused by, eg, inflammation, **ischaemia, or mechanical deformation**) **If radicular pain coexists with radiculopathy, deafferentation pain may also contribute**	Lancinating, shocking, electric, **burning, sharp**, stabbing, and shooting. **Often** accompanied by dull background aching. Can also include paraesthesia and **dysaesthesia**	Pain radiating into the leg, in areas reminiscent of but not necessarily identical to dermatomes. Deep and cutaneous
Radiculopathy	**Lesion or disease of a nerve root or dorsal root ganglia. Associated with a conduction slowing or block**	Neurological deficits compatible with the innervation territory of the affected nerve **Radiculopathy can be pain-free but may be accompanied by radicular pain (painful radiculopathy)**	Neurological deficits in dermatomal or myotomal distribution

The areas updated by the working group are highlighted in bold.

### 3.2. Outcomes phase 1a: terminology

The working group agreed that the term “sciatica” is not accurate (ie, the sciatic nerve is typically not affected with spine-related leg pain), is used inconsistently (ie, no agreed definition or clinical criteria), and does not always help to make sense of pain for patients. However, the panel also acknowledged that the term “sciatica” has a historically strong hold in the wider clinical and research communities: It is well established in patient and public language (even if its use is not always in a neural context) and firmly embedded in the scientific literature (eg, it is Medical Subject Headings in leading search databases). For these reasons, and from a pragmatic point of view, the panel recognised that the term “sciatica” is likely to be retained by the wider public and research community.

Nevertheless, the panel recommended discouraging the term “sciatica” for use in clinical practice and research. If used, it should include further specification of what the term means. Clinically, such specification may include a more accurate explanation for patients (ie, that the source is in the back and that there is variability among presentations. For an example see https://www.youtube.com/watch?v=HJvNBYOKf64). In a research setting, further specification should include the use of more accurate case definitions (see below for recommended case definitions for specific spine-related leg pain). As a minimum, a description of specific clinical criteria used as case definition for the study population need to be provided.

### 3.2.1. Recommended case definitions of specific spine-related leg pain

The panel recommended the use of case definitions for specific spine-related leg pain, including the following:

(1) Somatic-referred pain
(2) Radicular pain without or with radiculopathy

The case definitions of somatic-referred pain, radicular pain, and radiculopathy have already been defined and described by Bogduk^6^ and have been incorporated into the IASP terminology.^30^ The working group in principle agreed with these case definitions and their descriptions. We collated the descriptions of each case definition and added several clarifying adjustments (Table 1, and text in italics below). Of note, the panel emphasised that differentiation of these specific case definitions needs to take place in the context of the broader clinical presentation and, ideally, a detailed clinical examination.

#### 3.2.1.1. Somatic-referred pain

Somatic-referred pain is caused by noxious stimulation of nerve endings in somatic structures but is perceived in regions innervated by nerves other than those innervating the site of noxious stimulation.^6,30^ Somatic referred pain may originate from discs, facet joints, and muscles, as per previous definition.^30^ *We have further added tendons, ligaments, fascia, and bones as potential sources of somatic-referred pain in the context of spine-related leg pain.* Indeed, these structures are nociceptively innervated^1,4,49^ and can produce referral patterns into the lower limb.^15,33^ The pain may be perceived as dull, aching, gnawing, or pressure. *We have also clarified that although somatic referred pain mostly presents in the gluteal and thigh area, it may occasionally refer to the lower leg but rarely to the foot*.^15,33^ *We have further clarified that somatic referred pain is usually perceived as deep but rarely cutaneous*. Importantly, somatic referred pain needs to be distinguished from visceral referred pain, which may also refer to the limbs and particularly to the gluteal or groin areas in the lower limbs.^27^ In this case, a thorough clinical history will be important to understanding the nature of the focal and referred pain.

#### 3.2.1.2. Radicular pain without radiculopathy

Radicular pain is likely associated with *hyperexcitability* and ectopic discharges of dorsal roots or dorsal root ganglia. The hyperexcitability and ectopic discharges may be due to inflammatory irritation, *ischaemia, or mechanical deformation* of neural structures or a combination of these and their downstream mechanisms (eg, inflammation).^61,62^

The pain may be described as shocking, lancinating, electric, stabbing, or shooting and is often accompanied by a dull background ache.^51,65^ *We have further added sharp and burning as potential pain descriptors* as these are frequently reported by patients with lumbar radicular pain.^51^ *With radicular pain, the pain may be spontaneous or evoked, for instance, by certain movements of the spine or leg*. In addition to pain, patients may experience paraesthesia and *dysaesthesia* such as tingling, a “woolen feeling,” or “a block of ice.”

The pain can be felt deep or cutaneous and may radiate into the leg in areas reminiscent of, but not necessarily identical to, dermatomes.^51^ The working group further agreed to remove the original description of “band-like” location of the pain^6,30^ which seemed sufficiently covered by “areas reminiscent of, but not necessarily identical to, dermatomes.”

#### 3.2.1.3. Radicular pain with radiculopathy

Radicular pain may occur in association with a radiculopathy.^30^ Radiculopathy is caused by a lesion or disease of a nerve root or dorsal root ganglion. Radiculopathy is clinically characterised and defined by neurological deficits (eg, dermatomal hypoesthesia or anaesthesia, myotomal weakness, or reduced or absent reflexes) and may or may not coexist with pain. These neurological deficits are caused by neural conduction slowing or block of small or large nerve fibres or actual axotomy. Whereas true neurological deficits are usually stable, fluctuations for instance with positional changes have been described.^60^ Such fluctuations may be related to ischaemic rather than demyelinating conduction block.

Although painless radiculopathy certainly exists (eg, pure motor radiculopathy or mixed sensory and motor radiculopathy without pain), radiculopathy may occur in association with radicular pain, also referred to as painful radiculopathy.^30^ The similarities between radicular pain with and without radiculopathy are evidenced by largely comparable pain severity and pain characteristics in those presentations.^72^ Occasionally, people with radiculopathy may also have somatic referred pain.^30^ One example is a person with residual neurological deficits 2 years after acute onset, who now reports somatic referred pain in the buttock area.

#### 3.2.1.4. Recommendation of an umbrella term encompassing these 3 specific case definitions

The panel agreed that it would be useful to have an umbrella term that includes these 3 specific case definitions. This was considered important both for clinical and research purposes to distinguish limb symptoms originating from structures in the back from those originating from nonspinal structures (eg, somatic-referred pain from the hip and vascular leg pain). Unlike sciatica which linguistically alludes to neural problems, this term should encompass all 3 case definitions of somatic-referred pain, radicular pain and painful radiculopathy.

The following 2 terms were proposed by the working group:

(1) Spine-related leg pain
(2) Back-related leg pain

These 2 terms were taken to a wider consultation with the NeuPSIG membership (see 3.3).

### 3.3. Outcomes phase 1b: Neuropathic Pain Special Interest Group membership consultation

A consultation was sent out to n = 745 NeuPSIG members by email on the 8th of March 2022, and a reminder email was sent on the 22nd of March 2022. Members were asked to complete a short survey (Appendix 2, available as supplemental digital content at http://links.lww.com/PAIN/B813) where they were given some background about the working group and its objectives and asked the following questions:

We would like to ask your opinion on which of these proposed terms you would prefer:

(1) Spine-related leg pain
(2) Back-related leg pain
(3) Neither of these 2 terms

**Figure 2.**
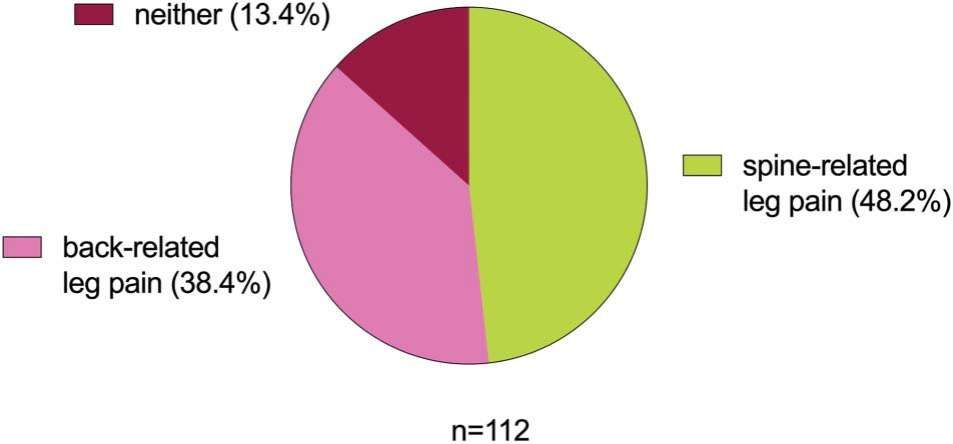
Results of the NeuPSIG membership terminology survey. Participants were invited to nominate their preference for the proposed terminologies. NeuPSIG, Neuropathic Pain Special Interest Group.

Overall, 112 members (15%) completed the survey. “Spine-related leg pain” was the preferred term (>48% of votes) (Fig. 2). However, the relatively high number of ratings for “back-related leg pain” and the fact that >13% prefer neither of these 2 terms highlight the ongoing controversy surrounding the terminology. In line with the preference of the working group, several comments by NeuPSIG members highlighted that spine-related leg pain has the advantage, that it can also be adopted for cervical conditions (ie, spine-related arm pain). Furthermore, spine-related leg pain was considered to be more specific, whereas back-related leg pain may include conditions unrelated to the spine, such as sacroiliac joint problems or piriformis syndrome. On the other hand, it was mentioned that back-related leg pain may be more adequate in lay language. Given the differing views, our survey's focus on NeuPSIG members alone, and the relatively small proportion of respondents, future initiatives should include a wider consultation on the appropriateness and usefulness of this term.

## 4. Phase 2: identification of neuropathic pain

It is well established that a proportion of patients with spine-related leg pain have neuropathic pain. Prevalence values range from 19% to 80% partly reflecting varying diagnostic criteria to identify neuropathic pain.^26^ The controversies surrounding the question whether spine-related leg pain, in particular radicular pain, is neuropathic, is reflected in the International Classification of Diseases (ICD)-11 coding system.^80^ “Sciatica” (ME84.3) and “lumbago with sciatica” (ME84.20) are listed under “symptoms, signs, or clinical findings of the musculoskeletal system.” By contrast, “radiculopathy,” “radicular pain,” and “nerve root and plexus compressions” are listed under “diseases of the nervous system.” Of note, the term chronic peripheral neuropathic pain includes “chronic painful radiculopathy.” As such, depending on the terminology used to define patients with spine-related leg pain (ie, sciatica or radicular pain or painful radiculopathy), ICD-11 coding could either suggest a nociceptive/musculoskeletal or a neuropathic condition for the same presentation. This highlights potential areas in operationalising the ICD-11 that may need revising or further clarification.

**Figure 3.**
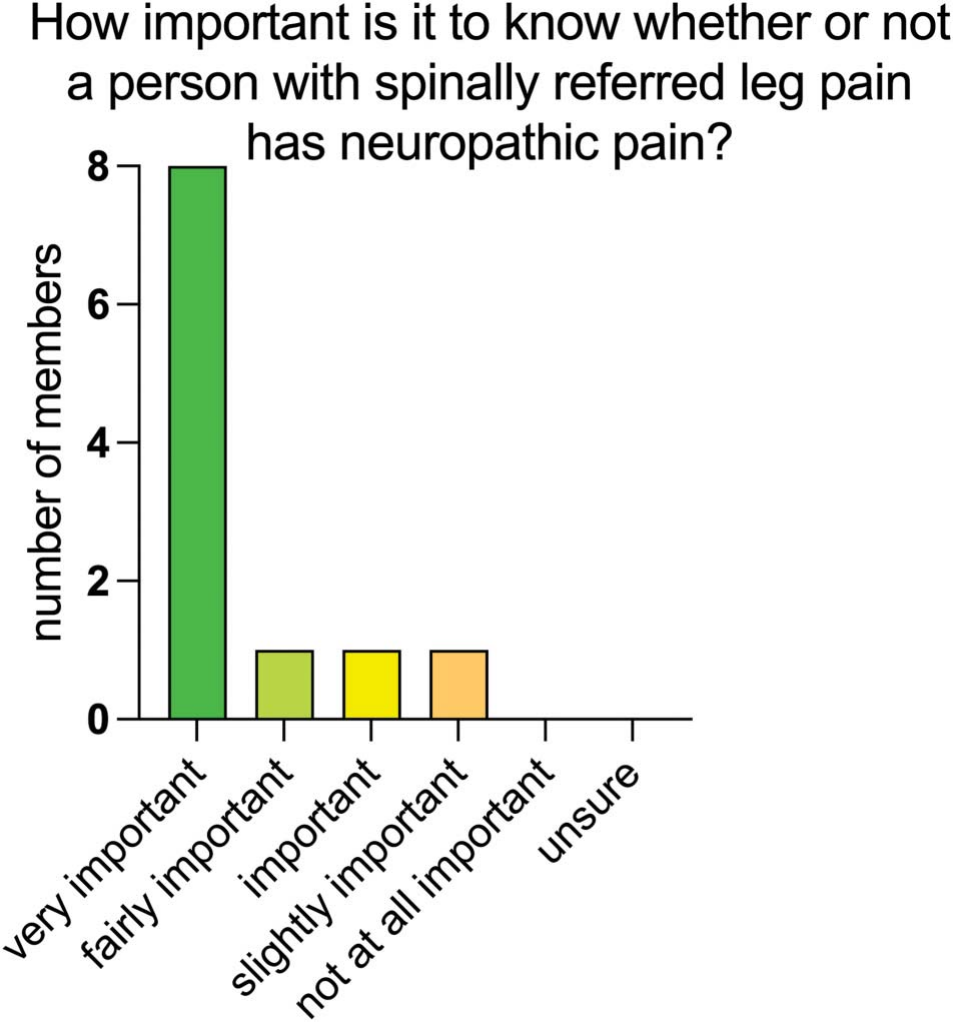
Panel ratings for the importance of identifying neuropathic pain in a person with spine-related leg pain.

In the premeeting survey sent to the working group, there was strong consensus that it is important to know whether or not a person with spine-related leg pain has neuropathic pain (Fig. 3). The most frequent reason given was that the information would guide treatment and management, including self-management.

The challenge, however, is to determine whether spine-related leg pain, in particular, radicular pain, is nociceptive, neuropathic, or mixed. Patients with radicular pain often describe their symptoms with seemingly neuropathic characteristics, such as electric shocks, shooting pain, tingling, and pins and needles. However, the frequent absence of obvious signs of a nerve lesion (eg, sensory loss of function and diagnostic tests), the absence of a dermatomal symptom distribution in 2 thirds of patients with radicular pain,^51^ and the frequent lack of a specific history indicating a neural lesion mean that many patients do not meet the diagnostic criteria for peripheral neuropathic pain outlined in the ICD-11 system.^64^ In the absence of sensory changes and magnetic resonance imaging (MRI) findings, patients with radicular pain would at best reach a classification of possible neuropathic pain according to the NeuPSIG neuropathic pain grading system.^17^ As such, identifying the presence of neuropathic pain in patients with spine-related leg pain can be challenging.

For Phase 2, the panel was tasked with the following activities:

(1) Discussing and reaching consensus on the classification of neuropathic pain for the specific terms recommended in objective 1 and
(2) Considering the need for any specification or adaptation of the current neuropathic pain grading system to accommodate these recommendations.

### 4.1. Classification of neuropathic pain for different subgroups of spine-related leg pain

**Figure 4.**
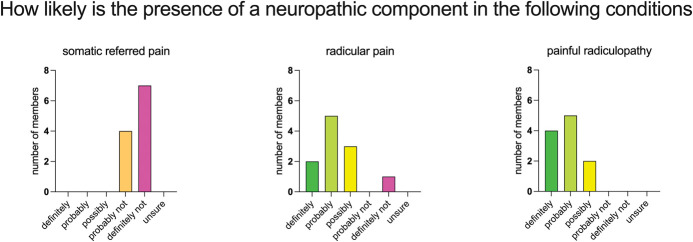
Panel ratings on the likely presence of neuropathic pain in various presentations of spine-related leg pain (6-point Likert scale from definitely to unsure).

In the initial survey of the working group members, there was broad agreement on the classification of 2 of the 3 terms regarding the likelihood of having neuropathic pain: somatic referred pain (probably not or definitely not) and painful radiculopathy (probably or definitely) (Fig. 4). The working group members concorded that neuropathic pain may be a feature in radiculopathy.

The panel discussed at length whether radicular pain should be classified as neuropathic pain or not, and initially, a consensus was not reached. One strategy posed to the panel to facilitate further discussion was to simply ask the fundamental question; “is there neuropathic pain or not in a person presenting with spine-related leg pain?” The fundamental question is posed agnostic to terminology and case definitions in the first instance, thereby bypassing the forced attribution of neuropathic pain to a specific term (eg, radicular). Such an approach does not compromise the application of the neuropathic pain grading system to clinical pain conditions but rather focuses on attributing the classification of neuropathic pain to the presentation rather than to a specific terminology.

By applying this strategy, the panel was able to reach consensus on the following recommendations:

#### 4.1.1. Somatic-referred pain

Somatic-referred pain does not meet the current grading system criteria for classification as neuropathic pain. The history (eg, reason for symptom onset such as muscle strain and pain descriptors) is not indicative of a nerve lesion, and diagnostic tests do not confirm the presence of a nerve lesion. Sensory signs may at times be present, but with varying borders and characteristics, and they do not follow clear neuroanatomical distributions. This does not preclude the coexistence of neuropathic pain in association with a comorbid pain condition (eg, a painful radiculopathy). In this case, although, neuropathic pain would not be attributable to somatic-referred pain but rather to the comorbid neuropathic condition.

#### 4.1.2. Radicular pain without radiculopathy

Radicular pain in the absence of a radiculopathy would not meet all the current grading scheme criteria for classification as neuropathic pain (see above for a lesion which is not detectable with sensory examination). This does not preclude the coexistence of nonneuropathic pain with other comorbid pain conditions (eg, somatic-referred pain).

#### 4.1.3. Radicular pain with radiculopathy

Radicular pain with radiculopathy (painful radiculopathy) meets the current grading system criteria for classification as neuropathic pain, correlating with the ICD-11 definition of neuropathic pain in painful radiculopathy. This does not preclude the coexistence of neuropathic pain in this case with other nonneuropathic pain, eg, somatic-referred pain. Radiculopathy with only somatic-referred pain would not meet the current grading scheme criteria for neuropathic pain.

### 4.2. Adaptation of the neuropathic pain grading system to spine-related pain

The neuropathic pain grading system^17^ was developed to assist clinicians and researchers in determining whether patients have neuropathic pain (or a combination of another pain type and neuropathic pain) and the level of confidence associated with that decision. Based on clinical and laboratory examination findings, patients are classified as having no neuropathic pain or possible, probable, or definite neuropathic pain.

The requirements for possible neuropathic pain are as follows:

(1) History of relevant neurological lesion or disease and
(2) Pain distribution neuroanatomically plausible.

The requirement for probable neuropathic pain is that pain is associated with sensory signs in the same neuroanatomically plausible distribution. Alternatively, possible neuropathic pain with the absence of cutaneous sensory signs but with the presence of a confirmed diagnostic test is also classified as probable neuropathic pain.

Definite neuropathic pain is reached if all the above criteria are present, plus a diagnostic test confirming a lesion or disease of the somatosensory nervous system explaining the pain.

It is recommended that the presence of probable or definite neuropathic pain prompt consideration of treatment according to the neuropathic pain treatment guidelines.^17^ Currently, in the context of spine-related leg pain, however, there is little evidence that neuropathic pain medications are effective.^24,57^ However, very few trials actually mandated the presence of neuropathic pain as an inclusion criterion,^52,79^ meaning we cannot preclude heterogeneity in case definitions, further confounding/complicating the interpretation of true efficacy. Other studies used underpowered (post hoc) subgroup analyses.^48,76^ Future research is required to determine whether neuropathic pain medications are effective in patients with spine-related leg pain with neuropathic characteristics and clarification of outcomes may in part be facilitated by the use of specific case definitions, as proposed in this paper. In the absence of clear evidence for efficacy in pharmacological treatment, targeted management strategies for people with neuropathic pain would still be distinct from those with nociceptive pain, for instance, related to explanations, education, and sense making. As such, the grading system classification has direct clinical implications for care recommendations including in spine-related leg pain.

The panel discussed the application of the neuropathic pain grading system in the context of spine-related leg pain, including the use of several case studies of patients to test operationalisation of the system. This discussion identified differences in the application of the grading system among panel members and highlighted that the further clarification on the interpretation of requirements for possible and probable neuropathic pain was needed in the context of spine-related leg pain. The following challenges and uncertainties to the interpretation of the current grading system were identified by the working group:

(1) The often insidious onset of spine-related leg pain with no clear temporal or spatial relationship with a nerve lesion and how to interpret that in relation to the “history of relevant neurological lesion or disease.”
(2) The absence of a clear dermatomal symptom distribution in about two thirds of patients with radicular pain^51^ and whether such pain presentation would still be considered as “neuroanatomically plausible.”
(3) The presence of neuropathic symptom descriptors such as electric shocks, shooting pain, tingling, and pins and needles or aggravating or alleviating factors suggestive of pain relating to a neurological lesion may be the only indications in patients with radicular pain. It remained unclear how much weight would be attributed to these symptoms, especially in the absence of a history of a neurological lesion.
(4) The frequent absence of sensory loss on bedside sensory examination. Sensory loss of function may in few people only be apparent on laboratory quantitative sensory testing, which may not be available in all clinical settings.^20,73^ However, using quantitative sensory testing delineation of borders is not possible, leaving uncertainty about the neuroanatomically plausible distribution of the sensory loss.
(5) The absence of frank sensory loss but presence of thermal hyperalgesia in patients with neuropathic pain/nerve injury, including patients with lumbosacral radiculopathy (∼33%)^2^ or mechanical allodynia in patients with lumbar radicular pain with or without radiculopathy.^9^ However, gain of function is not necessarily discriminative of neuropathic conditions, as it is also found in nociceptive conditions.^8,16^ A typical example is the widespread mechanical hyperalgesia reported in patients with nonspecific low back pain.^14^
(6) It was unclear to panel members how much weight would be given to “positive” sensory signs in the absence of sensory loss.
(7) Some patients have myotomal loss of function (weakness or reflex reduction) in addition to their pain (e.g., painful motor radiculopathy).^71^ This can be indicative of a nerve root lesion, but this is not considered in the current grading system.
(8) The questionable sensitivity of many confirmatory tests to detect nerve involvement in patients with spine-related leg pain. For instance, routine MRI displays about 30% false positives and false negatives^78^ and positional dependence seems to influence interpretation (eg, higher nerve root compression grades during standing than the conventional supine position).^53^

**Figure 5.**
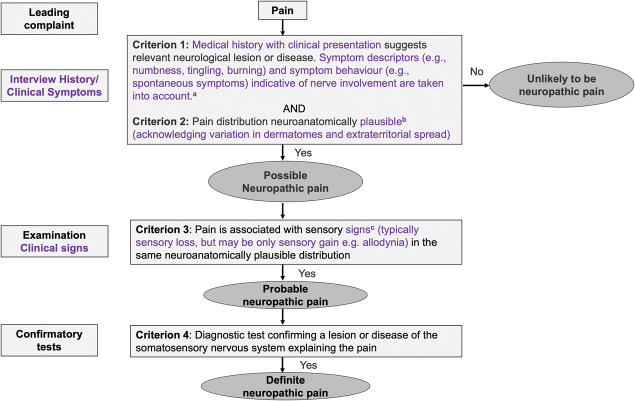
Flow chart of the panel recommendations on adaptations of the grading system for neuropathic pain in people with spine-related leg pain. Adaptations are highlighted in purple colour. Further clarification is provided in the text following each recommendation.

The panel, therefore, recommended the following adaptations to the neuropathic pain grading system in the context of spine-related leg pain (Fig. 5).

#### 4.2.1. Recommendation 1: possible neuropathic pain

The minimum requirements for possible neuropathic pain are as follows: (1) a medical history with clinical presentation suggestive of a neural lesion or disease (eg, surgery, trauma, neuropathic characteristics, or behaviour of symptoms) and (2) a neuroanatomically plausible pain distribution. Both criteria need to be fulfilled to reach the first level of “possible” neuropathic pain.

Below, we explain the specifications made to the grading system (Fig. 5) and their implementation.

##### a) Medical history with clinical presentation

The working group decided to clarify the meaning of “history.” Depending on medical or health speciality, the term “history” may be limited to temporal development of a presentation only or also include the full image of the clinical presentation. In the grading system, “history” refers to comprehensive information obtained both on temporal aspects as well as clinical presentation. In the case of radicular pain and painful radiculopathy, a specific cause or trigger for the onset of symptoms is often absent and symptoms may develop insidiously. Hence, a temporal relationship between the lesion or disease and the pain may not be apparent in some patients.^70^ Symptom descriptors, eg, pain descriptors, such as electric shocks, shooting, pins and needles, burning, and nonpainful sensations such as numbness and tingling, are suggestive, although not pathognomonic, of the presence of neuropathic pain^45,72,74^ and could be taken into account. The combination of several symptom descriptors such as those used in neuropathic pain questionnaires has been shown by some to have a high discriminant value.^5^ In the absence of a clear temporal relationship between the lesion or disease and pain, the presence of multiple pain descriptors of neuropathic nature taken together with the presence of characteristic symptom behaviour (eg, spontaneous pain, aggravating and alleviating factors, relationship between lower back and leg pain) can be sufficient to fulfill criterion 1 (history of relevant neurological lesion or disease). The importance of symptom descriptors was already mentioned in the article of the revised grading system,^17^ but we have now made this explicit by incorporating them into the grading flow chart.

##### b) Pain distribution neuroanatomically plausible

The pain distribution should be consistent with the innervation territory of the suspected nerve root lesion/disease, although it has to be acknowledged that dermatomal distributions vary^40^ and extraterritorial spread may occur.^51^ In addition, a dermatomal distribution may be difficult to establish in patients with deep pain alone. As such, pain beyond traditional dermatomal territories can still be considered neuroanatomically plausible in people with radicular pain with or without radiculopathy.

#### 4.2.2. Recommendation 2: probable neuropathic pain

The pain is associated with sensory signs in the same neuroanatomically plausible distribution (see above on pain distribution vs distribution of sensory signs). This can be probed through a clinical examination, eg, bedside sensory testing, which should provide supporting evidence for the suspected nerve root lesion for criterion 3 (Fig. 5) to be met.

c)The area of sensory changes may extend beyond or overlap the area of pain. Sometimes, nociceptive pain may also be accompanied by the loss of function. However, this often does not follow neuroanatomical borders, is not reproducible, and has mainly been reported with sensitive laboratory tests (eg, quantitative sensory testing) rather than with bedside sensory examinations.^21,41^

Sensory signs in the relevant neuroanatomical distribution are typically negative sensory signs, ie, partial or complete sensory loss, indicative of a nerve conduction slowing or block or axotomy, respectively. Loss of function may occur in large or small sensory nerve fibres.^20,54,72,74,75^ As such, assessment of touch, vibration, pinprick, and cold and warm sensitivity is crucial for the examination of large and small fibre function.^17^ Positive sensory signs, such as tactile allodynia^9^ and thermal hyperalgesia,^2^ have been documented in patients with lumbosacral radicular pain or radiculopathy. Sensory gain can mask sensory loss, making it difficult to detect the latter with currently available bedside sensory tests. With the revised neuropathic pain grading system, positive sensory signs in the absence of sensory loss are deemed sufficient to satisfy the classification of probable neuropathic pain, as long as they are in a neuroanatomically plausible distribution.^17^ The expert panel agreed, although not unanimously, that positive sensory signs do not weigh as strongly as an indicator of a nerve lesion compared with sensory loss. However, in the absence of high-quality data, no recommendation could be made whether positive sensory signs taken in isolation are sufficient to fulfill the criteria of probable neuropathic pain in the context of spine-related leg pain.

Although bedside sensory testing may not be sensitive enough to detect sensory signs in some patients, subtle sensory abnormalities were found on quantitative sensory testing.^72^ In the absence of sensory signs, but the presence of a diagnostic test confirming a lesion or disease of the somatosensory system (eg, MRI), the grading of probable neuropathic pain is still applicable after careful consideration of causality.^17^

A straight leg raise test, or a slump test, is commonly performed clinically to detect neural tissue components or contributions to spine-related leg pain. However, these tests have limited diagnostic performance when used in isolation.^77^ Rather than being diagnostic of radicular pain with or without radiculopathy, these tests may detect neural tissue mechanosensitivity (to load or movement) and, therefore, may provide information about gain of function.^63^ As such, it could be argued that responses to straight leg raise or slump testing can be taken into account as part of positive sensory signs in the adapted grading system (criterion 3). However, recent research suggests that these tests can be negative in patients with a clear nerve lesion or disease, particularly in the presence of loss of sensory function.^3,7^ In the upper limb, analogous neural provocation tests have been shown to be positive in patients with widespread or generalised hypersensitivity (eg, nonspecific neck arm pain,^55^ whiplash,^66^ and fibromyalgia^69^), that is nonneuropathic in nature. It, therefore, remains unknown whether and how much weight such tests should be given when grading neuropathic pain certainty.

Sensory signs may be accompanied by motor signs, eg, myotomal or reflex deficit relevant to the root lesion or disease.^20,37,70,71^ Although motor signs are not part of the examination of the somatosensory system, and, therefore, not a requisite for the determination of the presence of neuropathic pain, loss of motor function relevant to the patient's presentation may increase the suspicion of a nerve root lesion. The decision about whether the loss of motor function is relevant to the condition is derived from the collective of information triangulated throughout the examination.

#### 4.2.3. Recommendation 3: definite neuropathic pain

For the level of certainty for definite neuropathic pain, an objective diagnostic test is required to confirm the suspected lesion or disease of the somatosensory nervous system explaining the pain.

In case of radiculopathies, computed tomography, magnetic resonance imaging, or other imaging techniques can confirm the presence of nerve root compromise at the relevant spinal level (eg, nerve root compression, flattening of the nerve root, or nerve root displacement).^38,72,74^ However, limitations mentioned above (eg, high false positive and false negative rate and positional dependence) should be acknowledged. Electrodiagnostic studies may indicate large fibre function compromise but cannot assess small fibre compromise.

Confirmative objective diagnostic tests are rarely available in the nonspecialist environment (eg, primary care) or lower resource settings or may not be indicated at least initially (eg, in spine-related leg pain where serious pathology is not expected). However, patients may still fulfill all criteria for the classification of probable neuropathic pain, and treatment according to the neuropathic treatment guidelines in this context should be considered.^17^ Even if only the requirements for possible neuropathic pain are met, but a clinician has very strong suspicion for the presence of neuropathic pain (eg, relevant motor signs are apparent and fit the symptom image or a patient rates high on neuropathic pain screening tools), treatments targeting neuropathic pain may be appropriate and indicated where potential benefits for the individual outweigh risks. This interpretation of the clinical grading system will enable clinicians in lower resourced settings to make appropriate clinical decisions when indicated.

### 4.3. Future directions in research

The recommendations from this working group serve as a foundation for future scientific efforts. The following have been identified as priority areas:

(1) Agreement on diagnostic criteria for each case definition (somatic-referred pain, radicular pain with or without radiculopathy) to standardise participant selection for clinical studies and facilitate pooling of data.
(2) Incorporation of case definitions and the adapted neuropathic pain grading system in future studies of spine-related leg pain to facilitate improved patient profiling and translation to clinical settings.
(3) Exploration of the value of case definitions and the adapted neuropathic pain grading system as stratification tools to identify groups of people for whom specific interventions may be appropriate.
(4) Operationalising the recommendations into trial design and monitoring outcomes to evaluate whether these recommendations help to improve trial quality and ability to interpret true efficacy for people with spine-related leg pain

## 5. Conclusion

In conclusion, the working group has revised the terminology of spine-related leg pain and its specific case definitions. The panel recommends discouraging use of the term “sciatica” in clinical practice and research; instead, accurate case definitions should be used. The panel, with support from the NeuPSIG membership consultation, proposes the term of “spine-related leg pain” as an umbrella term to include the case definitions of somatic-referred pain and radicular pain with and without radiculopathy. This term may help differentiate pain originating from structures in the back from those originating from nonspinal structures (eg, somatic-referred pain from the hip).

The panel also proposed an adaptation of the neuropathic pain grading system in the context of spine-related leg pain, to facilitate the identification of neuropathic pain and initiation of specific management in this patient population.

Overall, the panel highlighted that the grading system is ideally applied within and augmented by the clinical work up, rather than considered in isolation. Furthermore, the proposed recommendations should be seen as a starting point that will likely need to be reviewed and revised as new knowledge arises and a more definitive understanding emerges. Table 2 summarises the recommendations and recommended actions in research.

**Table 2:** Recommendations and recommended actions for research.

Working group recommendation	Recommended actions in research settings
Avoid the term “sciatica” without further specification	Use “spine-related leg pain” as an umbrella term instead Use the specific case definitions of “somatic-referred pain,” “radicular pain,” and “radicular pain with radiculopathy” if specific patient population(s) are included
In the absence of agreed diagnostic criteria for spinally referred leg pain (including “sciatica”), provide a case definition with inclusion/exclusion criteria for the studied patient population in detail	Avoid general statements such as, “people with clinically diagnosed “sciatica” were included” without further specification. Instead, provide a case definition including exact details of specific clinical criteria used to select the patient population. Criteria should be sufficiently clear so that the study can be replicated with specific cohorts or populations and that inferences about generalisability of outcomes to clinical practice can be made. Example: Avoid: unclear definitions such as “back and leg pain” or “neurological evidence of nerve root affection.” Do: provide specific details such as “neurological evidence of relevant nerve root affection as evidenced by dermatomally reduced light touch or pinprick sensation OR reduced myotomal muscle strength lower than Medical Research Council scale M5 OR absent or reduced related lower-limb reflexes” Studies may also use published clinical diagnostic models such as that proposed by Stynes et al.* In this instance, the minimum required sum score and resulting minimum mean predicted probability should be reported
Use case definitions for specific spine-related leg pain	Report whether the study population includes one or all of the following: (1) Somatic-referred pain (2) Radicular pain (without radiculopathy) (3) Radicular pain with radiculopathy and how these were defined, preferably using the proposed terminology in this position paper If several case definitions are included, it is recommended that the percentage of each case definition and how they were defined are reported
Define and report the certainty of neuropathic pain	Use the adapted neuropathic pain grading system (Fig. 5) to identify and provide information on the presence and certainty of neuropathic pain Report the percentage of unlikely, possible, probable, and definite neuropathic pain If only participants with neuropathic pain are included, use the adapted neuropathic pain grading system as part of the inclusion criteria and specify which level of certainty was used as the cut-off

* Stynes et al.^68^

## Conflict of interest statement

A. B. Schmid receives grant funding from charities (Wellcome Trust, Medical Research Foundation, Versus Arthritis), government (UKRI, NIHR), and industry (ONO pharmaceutical). She receives lecture fees for postgraduate teaching in peripheral neuropathies and neuropathic pain. B. Tampin receives grant funding from the Sir Charles Gairdner Hospital and Osborne Park Health Care Group Research Advisory Committee and the Charlies Foundation for Research. H. Slater receives grant funding from the Australian Government (Commonwealth), the NH&MRC (MRFF), and Government of WA, Department of Health. R. Baron receives grant support from EU Projects: “Europain” (115007). DOLORisk (633491). IMI Paincare (777500). German Federal Ministry of Education and Research (BMBF): Verbundprojekt: Frühdetektion von Schmerzchronifizierung (NoChro) (13GW0338C). German Research Network on Neuropathic Pain (01EM0903). Pfizer Pharma GmbH, Genzyme GmbH, Grünenthal GmbH, Mundipharma Research GmbH und Co. KG., Novartis Pharma GmbH, Alnylam Pharmaceuticals Inc., Zambon GmbH, Sanofi-Aventis Deutschland GmbH. He received speaker fees from Pfizer Pharma GmbH, Genzyme GmbH, Grünenthal GmbH, Mundipharma, Sanofi Pasteur, Medtronic Inc. Neuromodulation, Eisai Co.Ltd., Lilly GmbH, Boehringer Ingel-heim Pharma GmbH & Co. KG, Astellas Pharma GmbH, Desitin Arzneimittel GmbH, Teva GmbH, Bayer-Schering, MSD GmbH, Seqirus Australia Pty. Ltd, Novartis Pharma GmbH, TAD Pharma GmbH, Grünenthal SA Portugal, Sanofi-Aventis Deutschland GmbH, Agentur Brigitte Süss, Grünenthal Pharma AG Schweiz, Grünenthal B.V. Niederlande, Evapharma, Takeda Pharmaceuticals International AG Schweiz, Ology Medical Education Netherlands. Ralf Baron received consultancy fees from Pfizer Pharma GmbH, Genzyme GmbH, Grünenthal GmbH, Mundipharma Research GmbH und Co. KG, Allergan, Sanofi Pasteur, Medtronic, Eisai, Lilly GmbH, Boehringer Ingelheim Pharma GmbH&Co.KG, Astellas Pharma GmbH, Novartis Pharma GmbH, Bristol-Myers Squibb, Biogenidec, AstraZeneca GmbH, Merck, Abbvie, Daiichi Sankyo, Glenmark Pharmaceuticals S.A., Seqirus Australia Pty. Ltd, Teva Pharmaceuticals Europe Niederlande, Teva GmbH, Genentech, Mundipharma International Ltd. UK, Astellas Pharma Ltd. UK, Galapagos NV, Kyowa Kirin GmbH, Vertex Pharmaceuticals Inc., Biotest AG, Celgene GmbH, Desitin Arzneimittel GmbH, Regeneron Pharmaceuticals Inc., Theranexus DSV CEA Frankreich, Abbott Pro-ducts Operations AG Schweiz, Bayer AG, Grünenthal Pharma AG Schweiz, Mundipharma Research Ltd. UK, Akcea Therapeutics Germany GmbH, Asahi Kasei Pharma Corporation, AbbVie Deutschland GmbH & Co. KG, Air Liquide Sante Inter-national Frankreich, Alnylam Germany GmbH, Lateral Pharma Pty Ltd, Hexal AG, An-gelini, Janssen, SIMR Biotech Pty Ltd Australien, Confo Therapeutics N. V. Belgium. N. B. Finnerup Outside the submitted work, NBF reports personal fees from Almirall, NeuroPN, Novartis Pharma, Vertex, and Nanobiotix and has undertaken consultancy work for Aarhus University for Biogen, Merz, and Confo Therapeutics, outside the submitted work and has received a grant from IMI2PainCare an EU IMI 2 (Innovative Medicines Initiative) public–private consortium and the companies involved are: Grünenthal, Bayer, Eli Lilly, Esteve, and Teva, outside the submitted work. P. Hansson declares no conflict of interest. K. Konstantinou declares no conflict of interest. C. Lin declares no conflict of interest. C. Price declares no conflict of interest. B. H. Smith was lead clinician for Chronic Pain for the Scottish Government (2014-2021) and receives grant funding from the Scottish Government, Eli Lilly, and UKRI/Versus Arthritis (not directly related to this work). A. Hietaharju received payment for lectures from Pfizer, Teva, Sanofi, and Lundbeck. Aki Hietaharju was the Chair of the NeuPSIG Executive Committee (2020-2022). J. Markman receives grant support from the National Institutes of Health. J. Markman received consultancy fees from Grunenthal, Merck AG, Eliem, Pfizer, Tremeau, Swan Bio, Nektar, Editas, Grunenthal, Biogen, Lilly, and Lateral Pharma. John Markman has served on Data Safety Monitoring Boards for Regenacy, Tonix, and Novartis pharmaceuticals. John Markman has equity in Yellowblack corporation and has served on the boards of Flowonix Corporation and the North American Neuromodulation Society.

## Appendix A. Supplemental digital content

Supplemental digital content associated with this article can be found online at http://links.lww.com/PAIN/B813.
